# Wake and break: deciphering the epigenetic regulation of dormancy onset and release in trees

**DOI:** 10.1093/plcell/koag069

**Published:** 2026-03-14

**Authors:** Pablo González-Suárez

**Affiliations:** Assistant Features Editor, The Plant Cell, American Society of Plant Biologists; Department of Developmental Genetics, Centre for Plant Molecular Biology (ZMBP), Eberhard Karls University, Tuebingen D-72076, Germany

Every year, organisms around the globe experience the arrival of winter. Unlike humans, plants do not warm themselves with layers of clothing and a cup of hot chocolate. Instead, they leverage physiological and developmental strategies to cope with the cold winter days. In particular, trees go through a yearly growth-dormancy cycle that ensures their survival during the frostier season (Fig. 1). In late summer, the shortening of days induces growth cessation. Shoot apical meristems (SAMs)—that is, stem cell niches that produce leaves during spring—slow their growth and form buds: protective structures that become dormant during autumn. Through dormancy, buds enter a state of mitotic quiescence that protects SAMs from the harsh environmental conditions. Prolonged cold exposure in the winter triggers dormancy release whereby buds regain their responsiveness to growth-promoting signals. Finally, in spring, warm temperatures promote bud break, and the new growth season begins.

**Figure 1:**
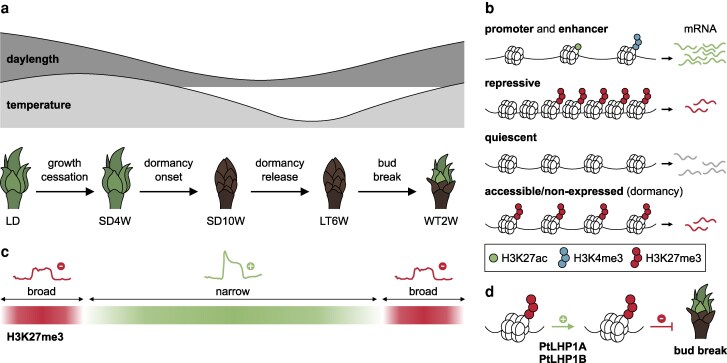
Genome-wide epigenetic reprogramming underlies the growth-dormancy cycle in hybrid aspen. a) Diagram illustrating the developmental stages of shoot apexes sampled by Li et al. (2026). The approximate temperature and daylength fluctuations associated with these stages are indicated above. LD, long days (active growth); LT6W, low temperature 6 weeks (ecodormancy); SD4W, short days 4 weeks (growth cessation); SD10W, short days 10 weeks (endodormancy); WT2W, warm temperature 2 weeks (bud break). b) Drawings illustrating major chromatin and epigenetic classes found in apexes throughout the growth-dormancy cycle. c) Temporal changes in H3K27me3 abundance and peak architecture. During dormancy (SD10W), narrow H3K27me3 are associated with noncanonical transcriptional activation. d) Model proposed by Li et al. (2026) whereby orthologs of LHP1 in hybrid aspen promote H3K27me3 maintenance, suppressing bud break. Figure credit: P. González-Suárez.

Strict genetic and hormonal control ensures that SAMs progress through this sequence in a timely manner. To date, several transcriptional regulators of dormancy onset and release have been identified, including phosphatidyl ethanolamine-binding proteins and MADS-box transcription factors. Chromatin modifications have also been proposed as an additional regulatory layer, and dynamic changes in the histone marks H3K27me3 and H3K4me3 are known to associate with dormancy regulators (Conde et al. 2019). However, genome-wide research into chromatin-based mechanisms is lacking in trees and 2 questions remain. How does chromatin accessibility influence seasonal development and what epigenetic mechanisms facilitate developmental transitions?

In their recent work, Li, Xu and coauthors **(Li et al. 2026)** tackled these questions from a multi-omic perspective. Focusing on hybrid aspen (*Populus tremula × P. tremuloides*), they set out to characterize the chromatin landscape throughout the bud growth-dormancy cycle. First, they sampled shoot apexes in a time-course experiment to study 4 key developmental transitions: (1) growth cessation, (2) dormancy onset, (3) dormancy release, and (4) bud break (Fig. 1). They then used this material to generate omics profiles aiming to capture 3 levels of regulation: transcriptional control (RNA-seq), chromatin accessibility (ATAC-seq), and histone modification (ChIP-seq). The latter revealed genome-wide changes in 3 epigenetic marks associated with negative (H3K27me3) and positive (H3K4me3 and H3K27ac) transcriptional regulation (Sato and Yamane 2024).

Through integrative analysis of the resulting datasets, the authors identified 4 major chromatin classes (Fig. 1). Two are active and show accessible chromatin and both H3K27ac and H3K4me3 marks. A third class is repressive, linked to lower gene expression and H3K27me3-marked regions. Lastly, a fourth quiescent class encompasses regions with no detectable histone modifications. This chromatin organization changes dynamically over time with genes switching between chromatin classes during the growth-dormancy cycle. Remarkably, many growth-related genes transition from active to repressive chromatin classes during dormancy establishment, concomitant with an accumulation of H3K27me3 marks. Conversely, numerous H3K27me3 peaks are lost during dormancy release and bud break.

Seasonal dynamics of histone modifications were consistent with our general understanding of their impacts on gene expression. H3K27ac was predominant in both distal and proximal open regions of highly expressed genes, and H3K4me3 was abundant in actively transcribed regions. H3K27me3, on the other hand, showed a more unexpected behavior (Fig. 1). Prior to growth cessation, H3K27me3 was associated with silent genes. However, after dormancy establishment, H3K27me3 correlated with neutral or even positive gene expression. This shift was associated with a change in H3K27me3 peak architecture, which narrows and sharpens during dormancy, suggesting a new noncanonical configuration of H3K27me3 that promotes transcription. Interestingly, H3K4me3 is also found in many of these genes, raising the possibility that co-occurrence of both marks is key for this positive effect on gene expression.

In an effort to identify the basis of the widespread epigenetic reprogramming of SAMs, the authors interrogate the potential role of LIKE HETEROCHROMATIN PROTEIN 1 (LHP1), a core Polycomb group protein involved in the maintenance and propagation of H3K27m3. Both of its homeologs in hybrid aspen, *PtLHP1A* and *PtLHP1B*, showed higher expression during dormancy. Additionally, knockout mutants and overexpression lines developed by the authors showed premature and delayed bud break, respectively, suggesting that *LHP1* is necessary for timely bud break and acts as a repressor of dormancy release in aspen (Fig. 1).

Epigenetic reprogramming is a key regulatory layer that underpins life-history transitions in plants. Li et al. (2026) provide a comprehensive characterization of the chromatin landscape during the seasonal growth of hybrid aspen, establishing the first atlas of chromatin in the seasonal growth cycle of trees. Notably, their work identifies dynamic changes in chromatin and epigenetic marks linked to specific developmental transitions, providing promising advances in the understanding of growth-dormancy cycles.

## Recent related articles in *The Plant Cell*

- Gao et al. (2024) identified an ortholog of ELONGATED HYPOCOTYL 5 in poplar that underpins dormancy establishment in response to short days.
- Kim et al. (2026) characterized a mechanism based on H3K27me3 deposition that mediates cold-induced growth retardation in *Arabidopsis thaliana*.
- Marcon et al. (2025) provided a comprehensive characterization of genome-wide transcriptional changes in buds and leaves during the growth cycle of aspen.

## Data Availability

No new data were generated or analysed in support of this research.

## Dive Curated Terms

The following phenotypic, genotypic, and functional terms are of significance to the work described in this paper:

HYPOCOTYL AmiGo: PO:0020100

h3k27ac CHEBI: CHEBI:85044

shoot apical meristem AmiGo: PO:0020148

LHP1 Gramene: AT5G17690
LHP1 Araport: AT5G17690
